# Breeding and study of two new photoperiod- and thermo-sensitive genic male sterile lines of polyploid rice (*Oryza sativa* L.)

**DOI:** 10.1038/s41598-017-15241-8

**Published:** 2017-11-07

**Authors:** Xianhua Zhang, Bo Zuo, Zhaojian Song, Wei Wang, Yuchi He, Yuhua Liu, Detian Cai

**Affiliations:** 1 Faculty of Life Science, Hubei University, Wuhan, 430062 China; 2 Wuhan Polyploid Bio-Technology Co., Ltd, Wuhan, 430345 China

## Abstract

Male sterile lines play an important role in the utilization of heterosis. To explore and exploit the heterosis of polyploid hybrid rice, two photoperiod- and thermo-sensitive genic male sterile lines of polyploid rice, PS006 and PS012, were bred via chromosome doubling, complex hybridization and self-breeding. The characteristics of these two lines, including the agronomic traits, growth, development, fertility transformation and combining ability, were investigated. Both lines had good agronomic characteristics and flowering habits, a high outcrossing rate, obvious fertility alterations and good combining abilities. Their hybrids showed strong heterosis and great potential for increasing rice productivity and quality. The new polyploid rice photoperiod- and thermo-sensitive genic male sterile lines will provide material for further research into polyploidy and hybrid vigour in rice and promote the exploitation of polyploid hybrid rice.

## Introduction

Rice (*Oryza sativa* L.) is one of the world’s three major food crops and represents an important component of the world’s food supply. Generating hybrid rice, which presents a higher grain yield than inbred rice varieties, is one of the most important applications of heterosis in agriculture^1–3^. Rice is a self-pollinating crop, and nearly all traditional rice cultivars are inbred lines. Since the 1970s, hybrid seed production has mainly used two-line or three-line hybrid systems^4,5^. The three-line system is based on a cytoplasmic male sterile (CMS) line, a restorer line (to produce F_1_ hybrid seeds) and a CMS maintainer line (to maintain the CMS line)^6,7^. The three-line hybrid rice, which is referred to as “The Second Green Revolution”, has played an important role in promoting rice production. The two-line hybrid system is based on environmentally sensitive genetic male sterility^5,8^, and it usually uses photoperiod- and thermo-sensitive genic male sterile (PTGMS) lines as maternal parents to produce hybrid seeds. PTGMS lines are sterile under restrictive conditions (high temperatures and long days) but become fertile under permissive conditions (low temperatures and short days)^9,10^. The two-line hybrid rice further promotes increases in rice yield. However, rice is diploid and has a small genome and DNA content relative to polyploid species, such as wheat. The demand for increased rice yield and quality is high, and the limited genetic resources of diploid cultivated rice have hampered improvements in hybrid rice breeding^11^. Therefore, new ways or methods are needed to address this dilemma. A review of the trends in crop evolution suggests that the use of the double advantages of wide crossing and polyploidization to increase the number of rice genomes, raise the ploidy level and breed new rice varieties represents a new pathway for rice breeding^12^. Hybrids and polyploids (whole genome duplication) are common in plants and animals^13^. Both hybridity and induced polyploidy are potent methods for increasing the biomass, yield and resistance^14^. The special agronomic traits of polyploid rice, such as the large grain size and weight, strong stem and long panicles, are of particular interest for rice breeders^15–18^. Compared with diploid hybrids, polyploid rice hybrids present certain biological advantages, such as greater adaptability and yield potential, that are attracting the attention of rice researchers^16,19–21^. However, autotetraploid rice has many unfavourable traits, especially low fertility, which has the largest negative impact on polyploid rice breeding. After years of efforts, this problem has been resolved with the polyploid meiosis stability (PMeS) tetraploid rice line, which was successfully bred by our research group in 2007^22^. PMeS lines have stable meiosis and a high seed-setting rate (more than 70%). Moreover, their hybrids also present these characteristics and can have even higher seed-setting rates of 80–90%^23,24^. In recent years, other new tetraploid rice lines with high seed setting (>80%) have also been bred by professor Liu’s research group, and two new lines of these have been registered for the “Protection for New Varieties of Plants in China”^14,25^. Successful breeding of high-fertility tetraploid rice has promoted the development of polyploid rice breeding. Therefore, breeding polyploid rice male sterile lines and studying the heterosis of polyploid rice are important for the use of polyploid rice. Until now, the three-line system of polyploid rice has been researched by Tu *et al*.^26^. Here, we report the breeding procedure, agronomic traits, fertility transformation and combining ability in two new PTGMS lines of polyploid rice. Our study provides new germplasm for rice breeding and lays the foundation for studying the two-line hybrid system of polyploid rice.

## Results

### Biological characteristics of PS006 and PS012

The chromosome numbers in the root tips of PS006 and PS012 plants are 2n = 4*x* = 48.

**Figure 1:**
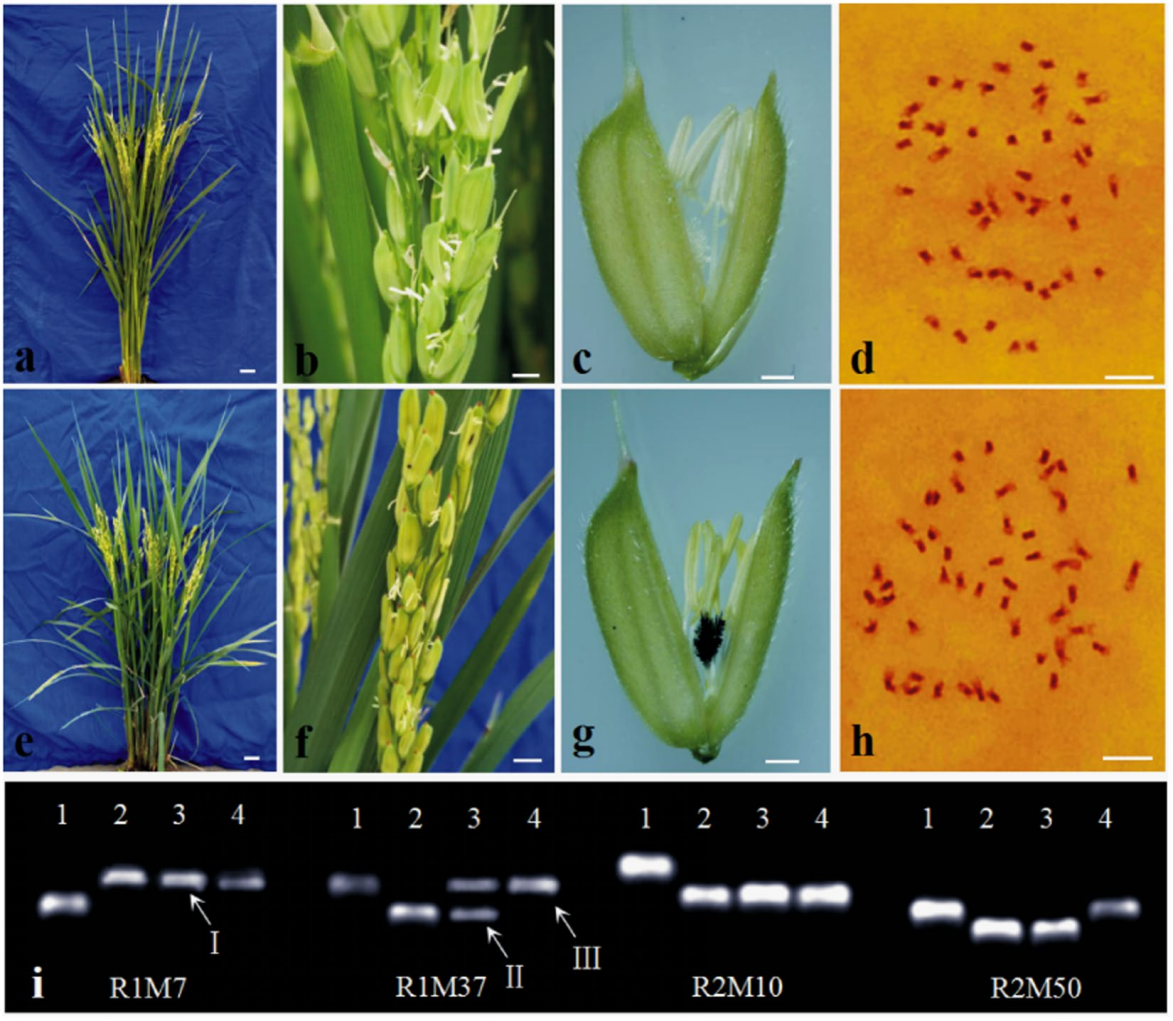
Morphological and chromosome traits. (**a–d**) Morphological traits and chromosomes of PS006. (**a**) Plant. (**b**) Spikelet. (**c**) Floret. (**d**) Chromosomes, 2n = 4*x* = 48. (**e–h**) Morphological traits and chromosomes of PS012. (**e**) Plant. (**f**) Spikelet. (**g**) Floret. (**h**) Chromosomes, 2n = 4*x* = 48. (**i**) Polymorphism of PS006 and PS012 based on PCR amplification using 19 InDel markers. This figure shows the results of four markers (R1M7, R1M37, R2M10 and R2M50). (1) Nipponbare (*O. sativa* ssp. *japonica*). (2) 9311 (*O. sativa* ssp. *indica*). (3) PS006. (4) PS012. Arrow 1 indicates *indica* homozygous genotype II. Arrow 2 indicates *indica-japonica* homozygous genotype IJ. Arrow 3 indicates *japonica* homozygous genotype JJ. *Bars* = 5 cm in (**a**) and (**e**), 5 mm in (**b**) and (**f**), 1 mm in (**c**) and (**g**), and 5 μm in (**d**) and (**h**).

PS006 is a tetraploid *indica* line derived from *indica-japonica* hybrid progenies. The gene frequencies of *indica* (F_i_)/*japonica* (F_j_) determined using InDel molecular markers were 0.84 and 0.16, respectively. The line had a comparatively compact plant type, straight flag leaves, large panicles, long oval grains, white awns, and large and white stigmas (Fig. 1a–d). These traits were stable in both Hainan and Wuhan. However, other agricultural characteristics, such as the plant height and tillering capacity, varied, with the plants usually growing taller in Wuhan (Table 1).

**Table 1:** Agronomic traits of po lyploid rice PTGMS lines.

Lines	Plant height (cm)	No. of tiller	Spikelet length (cm)	Grain length/width (cm)	Awn length (cm)	Stigma length (mm)	Anther length (mm)	Awn color	Stigma color
PS006 (Hainan)	76.10 ± 3.54	10.20 ± 1.53	22.5 ± 3.44	0.90 ± 0.11/0.33 ± 0.06	0.40 ± 0.13	1.40 ± 0.06	2.80 ± 0.56	white	white
PS006 (Wuhan)	98.63 ± 5.66	14.79 ± 2.31	26.54 ± 3.98	0.90 ± 0.06/0.33 ± 0.05	1.02 ± 0.74	1.41 ± 0.03	2.79 ± 0.60	white	white
PS012 (Hainan)	66.55 ± 5.12	12.00 ± 1.06	18.6 ± 2.78	0.85 ± 0.20/0.33 ± 0.02	0.20 ± 0.04	1.10 ± 0.02	3.00 ± 0.39	red	purple
PS012 (Wuhan)	91.45 ± 7.31	16.91 ± 3.15	22.06 ± 3.57	0.85 ± 0.16/0.33 ± 0.03	0.54 ± 0.12	1.11 ± 0.05	3.00 ± 0.22	red	purple

PS012 is a tetraploid *indica* line derived from inter-subspecific hybrids. The gene frequencies of *indica* (F_i_)/*japonica* (F_j_) were 0.82 and 0.18, respectively. The line showed a compact plant type with straight flag leaves, and it had dark green leaves, red awns and purple stigmas, which obviously differed from the characteristics of PS006 (Fig. 1e–h). Compared with the characteristics of PS006, the other agricultural characteristics included shorter plants but better tillering capacity (Table 1).

### Flowering habits of PS006 and PS012

**Figure 2:**
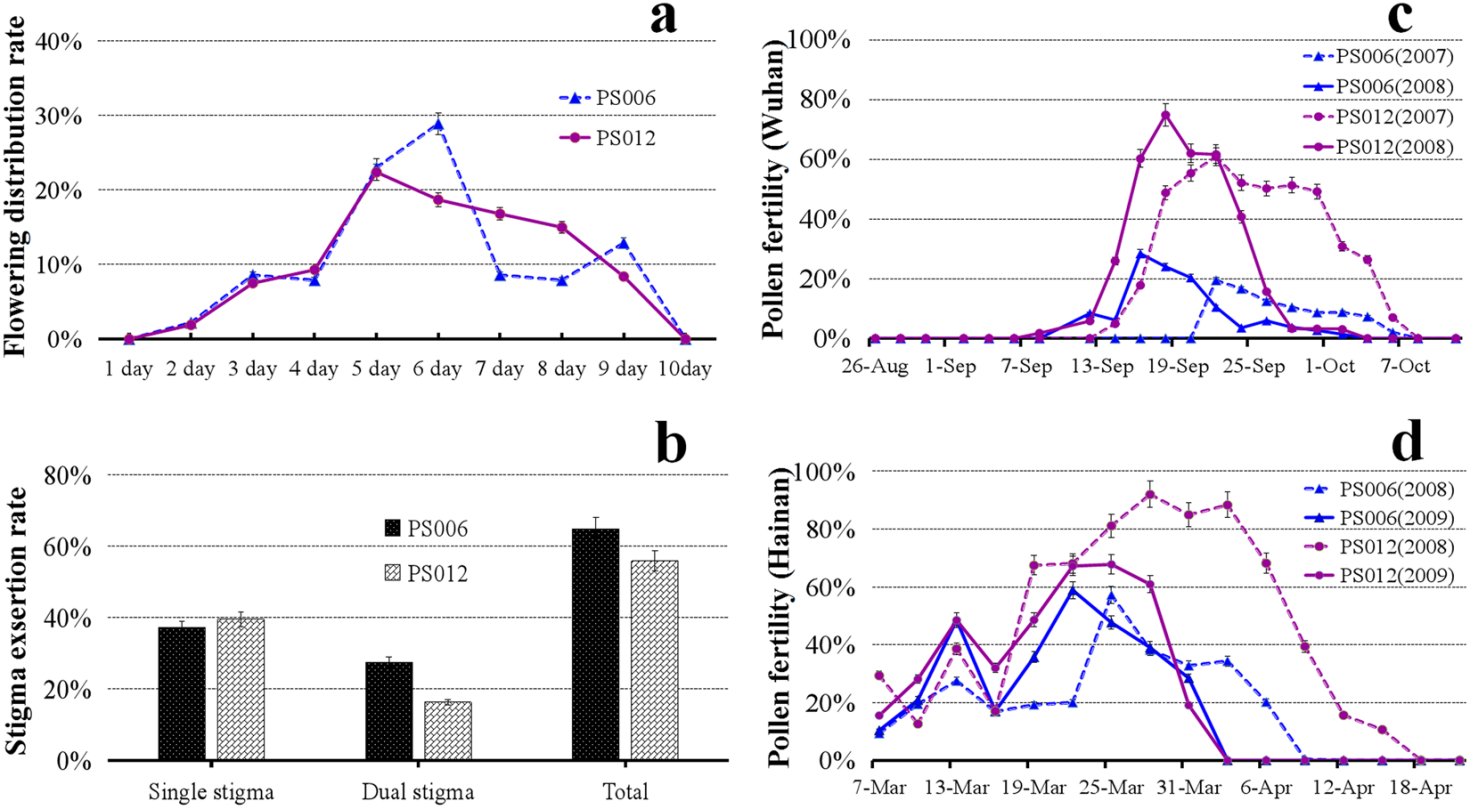
Flowering habits and pollen fertility characteristics. (**a** and **b**) Flowering habits of PS006 and PS012. (**a**) Flowering distribution of spikelets on a panicle of PS006 (blue dotted line) compared with PS012 (purple solid line). (**b**) Stigma exsertion characteristics of PS006 (black) and PS012 (white) grown under natural conditions. (**c** and **d**) Characteristics of pollen fertility in Wuhan and Hainan. (**c**) Changes in pollen fertility of PS006 (blue) and PS012 (purple) in 2007 (dotted line) and 2008 (solid line) in Wuhan. (**d**) Changes in pollen fertility of PS006 (blue) and PS012 (purple) in 2008 (dotted line) and 2009 (solid line) in Hainan.

The flowering duration of one spikelet and the flowering distribution of spikelets on a panicle were investigated to clarify the flowering habits of the PTGMS lines PS006 and PS012. Both lines showed good panicle uniformity and concentrated flowering periods. The blossoming in a single ear continued for 7–8 d. The full-bloom stage appeared 6 and 5 d after flowering for PS006 and PS012, respectively (Fig. 2a). The flowering time of one spikelet lasted 1.3–4.05 h for PS006 and 1.25–4.15 h for PS012.

Stigma exsertion is an important trait that contributes to seed production in hybrid rice. Both PS006 and PS012 had good exsertion rates and similar single stigma exsertion rates. However, the double stigma exsertion rate of PS006 was higher than that of PS012 (Fig. 2b), thus indicating the PS006 was more conducive to hybrid seed production.

### Characteristics of fertility alteration of PS006 and PS012

In both Hainan and Wuhan, PS006 and PS012 showed fertility alteration characteristics, and the stage sowing experiment showed similar results (Fig. 2c and d and Table 2). When planted in Wuhan, the two male sterile lines had white and small anthers at the heading stage and displayed complete male sterility before September under a photoperiod of 12.5–14.0 h and a temperature of 25–34 °C. In mid-September, which presented a photoperiod of 10.9–12.3 h and a temperature of 21–30 °C, pollen fertility began to recover. During this period, the lines produced few seeds by self-pollination. After October, the lines reverted to infertility. The recovery periods of PS006 and PS012 differed and presented differences among years. When planted in Hainan, a region in southern China that is warmer and suitable for rice growing in winter, PS006 and PS012 were fertile before April (a photoperiod of 11.0–12.5 h and a temperature of 21–28 °C), and both had good seed-setting rates. After April (a photoperiod of 11.0–12.5 h and a temperature of 24–29 °C), the pollen gradually became sterile. The accurate recovery period also showed differences among years. These phenotypes were observed consistently from 2008–2016 in both locations, suggesting that male sterility may be controlled by both temperature and photoperiod.

**Table 2:** The fertility performance of PS006 and PS012 for different heading-date.

Areas	Sowing-date (Y–M–D)	PS006			PS012		
		Heading-date (Y-M-D)	Pollen fertility rate (%)	Self-seeds rate (%)	Heading-date (Y-M-D)	Pollen fertility rate (%)	Self-seeds rate (%)
Wuhan	2008–05–26	08–08–05	0.00	0.00	08–08–01	0.00	0.00
	2008–06–05	08–08–12	0.00	0.00	08–08–10	0.00	0.00
	2008–06–15	08–08–18	0.00	0.27 ± 0.09	08–08–18	0.00	0.42 ± 0.25
	2008–06–25	08–08–24	0.00	0.00	08–08–24	0.00	0.32 ± 0.11
	2008–07–05	08–09–07	0.00	0.00	08–09–07	0.00	0.00
	2009–05–23	08–08–02	0.00	0.00	08–07–30	0.00	0.00
	2010–05–25	08–08–05	0.00	0.00	08–08–01	0.00	0.00
	2011–05–23	08–08–02	0.00	0.00	08–07–30	0.00	0.00
	2012–05–22	08–08–02	0.00	0.00	08–07–30	0.00	0.00
	2013–05–22	08–08–02	0.00	0.00	08–07–31	0.00	0.00
	2014–05–23	08–08–02	0.00	0.00	08–07–30	0.00	0.00
	2015–05–23	08–08–02	0.00	0.00	08–07–30	0.00	0.00
	2016–05–23	08–08–02	0.00	0.00	08–07–30	0.00	0.00
Hainan	2008–12–02	09–03–07	26.34 ± 12.98	11.03 ± 4.68	09–03–05	34.62 ± 12.20	18.91 ± 3.96
	2008–12–12	09–03–12	64.93 ± 14.52	36.44 ± 15.35	09–03–09	72.53 ± 11.51	46.79 ± 7.44
	2008–12–22	09–03–18	76.72 ± 16.31	47.60 ± 7.45	09–03–18	85.81 ± 13.58	68.15 ± 9.63
	2009–01–01	09–03–26	39.61 ± 9.26	19.63 ± 3.75	09–03–24	39.98 ± 10.86	21.43 ± 5.77
	2009–01–11	09–04–03	0.00	0.00	09–04–01	5.36 ± 1.04	0.00
	2010–12–02	09–03–07	35.73 ± 10.12	15.21 ± 3.75	09–03–05	58.67 ± 7.30	28.33 ± 5.14
	2011–12–02	09–03–07	29.31 ± 7.88	14.03 ± 2.91	09–03–05	49.51 ± 10.24	31.27 ± 2.90
	2012–12–02	09–03–06	58.34 ± 13.44	20.16 ± 5.08	09–03–05	64.55 ± 8.59	44.12 ± 7.01
	2013–12–02	09–03–07	43.52 ± 3.79	18.53 ± 6.94	09–03–05	56.32 ± 4.77	29.78 ± 6.33
	2014–12–02	09–03–07	51.26 ± 8.33	24.17 ± 4.02	09–03–05	70.59 ± 15.21	41.77 ± 8.31
	2015–12–02	09–03–07	36.57 ± 11.93	19.08 ± 5.32	09–03–05	68.18 ± 9.17	33.14 ± 5.09
	2016–12–02	09–03–07	60.57 ± 10.98	35.17 ± 8.54	09–03–05	75.14 ± 8.76	49.27 ± 3.93
	2017–12–02	09–03–07	48.30 ± 8.01	21.03 ± 7.60	09–03–05	59.43 ± 13.47	38.62 ± 5.16

The pollen fertility rate shown in this table is that at the initial heading stage.

**Figure 3:**
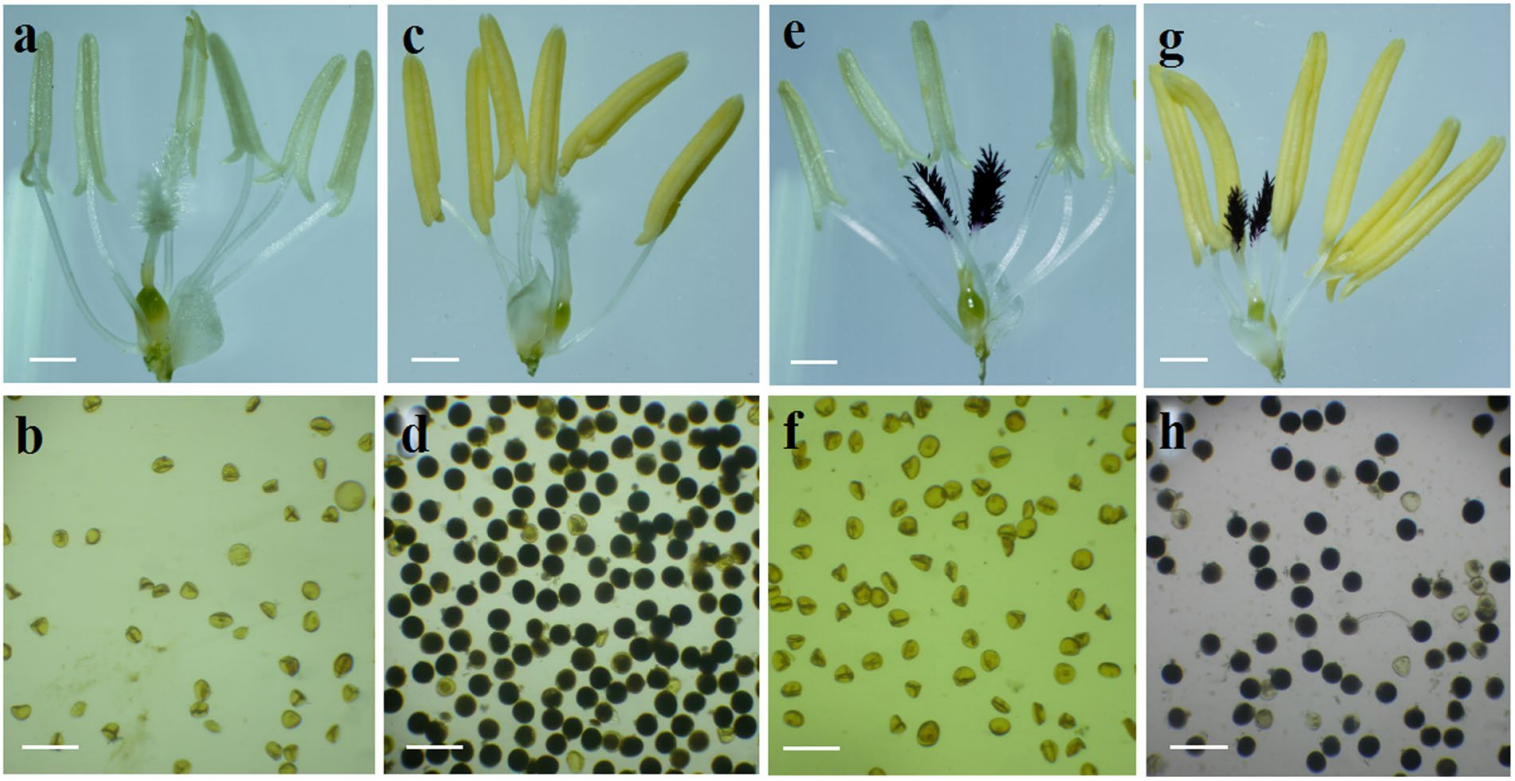
Characteristics of the anthers and pollen in fertile and sterile states. (**a** and **b**) Abnormal anther (**a**) and abortive pollen (**b**) of PS006 in the sterile stage. (**c** and **d**) Normal anthers (**c**) and pollen (**d**) of PS006 in the fertile stage. (**e** and **f**) Abnormal anther (**e**) and abortive pollen (**f**) of PS012 in the sterile stage. (**g** and **h**) Normal anthers (**g**) and pollen (**h**) of PS012 in the fertile stage. Bars = 0.5 mm in (**a**), (**c**), (**e**) and (**g**). Bars = 100 μm in (**b**), (**d**), (**f**) and (**h**).

The male sterile lines PS006 and PS012 also presented obvious characteristics of fertility transformation. In the different stages of fertility transformation, significant differences were observed in the anthers and pollen. In the sterile stage, the anthers were white and small (Fig. 3a and e), the pollen grains had irregular shapes, being triangular or prismatic under the microscope, and most of the mature pollen grains (>90%) were typical abortive pollen and did not result in pollination (Fig. 3b and f). These phenotypes indicated that pollen abortion of PS006 and PS012 primarily occurred at the microspore stage, suggesting that the two polyploid rice PTGMS lines were the sporophyte male sterile. In the fertile stage, PS006 and PS012 had normal plump yellow anthers and normal pollen grains (Fig. 3a–h).

### Fertility performance of PS006 and PS012 in a phytotron

PS006 and PS012 were grown in a phytotron at temperatures of 23, 24 and 28 °C and with two illumination times of 11.5 and 13.5 h. Temperature and illumination time had different effects on the fertility of PS006 and PS012 (Table 3). Significant correlations were observed between temperature and fertility for PS006 (F-value 163.777, P < 0.01) and PS012 (F-value 180.589, P < 0.01). The correlation coefficients between illumination time (F-value 20.833, P < 0.01) or photothermal interaction (F-value 22.642, P < 0.01) and fertility of PS012 were significant; however, none of these correlations were significant for PS006. The analysis illustrated that temperature was the main factor affecting fertility of PS006. Both temperature and illumination time affected fertility of PS012, although temperature was the major factor.

**Table 3:** The fertility performance of PTGMS lines in a phytotron.

Average temperature	Illumination time	PS006		PS012	
		Pollen fertility rate (%)	Self-seeds rate (%)	Pollen fertility rate (%)	Self-seeds rate (%)
23 °C	11.5 h	69.00 ± 6.67	20.15 ± 3.44	72.70 ± 6.77	35.3 ± 5.19
	13.5 h	56.20 ± 10.00	19.22 ± 2.89	69.9 ± 15.66	21.55 ± 3.37
24 °C	11.5 h	37.40 ± 4.57	2.03 ± 0.14	38.36 ± 6.38	6.77 ± 1.78
	13.5 h	26.25 ± 5.72	2.03 ± 1.49	29.22 ± 7.04	4.32 ± 1.99
28 °C	11.5 h	0.00	0.00	0.00	0.00
	13.5 h	0.00	0.00	0.00	0.00

### Sensitive stage, duration and sterile critical point of temperature (CPT) of fertility alteration

According to the methods of Mou^27^, the correlation between the daily mean temperatures at 0–21 d before heading and pollen fertility of PS006 and PS012 were analysed (Table 4). The results showed that specific stages of young panicle development in the two lines were sensitive to temperature. The sensitive stages were 6–15 d before heading, and the sensitive duration was 10 d.

**Table 4:** Correlation coefficients between daily mean temperatures of the 0–21 d before heading and pollen-fertility of PTGMS lines.

Days before heading	PS006	PS012	Days before heading	PS006	PS012
0–2	0.017	0.132	10–12	–0.444**	–0.499**
1–3	0.094	0.259	11–13	–0.381*	–0.546**
2–4	0.055	0.196	12–14	–0.353*	–0.533**
3–5	–0.075	0.002	13–15	–0.427*	–0.618**
4–6	–0.150	–0.062	14–16	–0.442*	–0.563**
5–7	–0.252	–0.260	15–17	–0.369*	–0.448*
6–8	–0.350*	–0.417*	16–18	–0.241	–0.214
7–9	–0.504**	–0.432*	17–19	–0.118	–0.005
8–10	–0.430*	–0.401*	18–20	–0.043	0.020
9–11	–0.400*	–0.403*	19–21	–0.004	0.047

*significant at P < 0.05, **significant at P < 0.01.

Regression analyses between the average daily mean temperature in the sensitive stages indicated that the CPT of fertility alteration were 23.6 °C and 24.4 °C for PS006 and PS012, respectively (Table 5). This implied that when the daily mean temperature during the sensitive stage was below 23.5 °C, both lines were fertile, and when it was above 24.5 °C, the lines were sterile. As a result, the lines could be used for hybrid seed production or reproduction according to the temperatures in the environments in which they are grown.

**Table 5:** Critical point temperature (CPT) of fertility alteration in PS006 and PS012.

Lines	Regression equation	F-value	Critical temperature
PS006	Y = 1.533–0.065*x*	9.427**	23.6 °C
PS012	Y = 2.073–0.085*x*	12.682**	24.4 °C

**Significant at P < 0.01.

### Outcrossing characteristics of PS006 and PS012

The polyploid rice PTGMS lines PS006 and PS012 were crossed with high seed-setting polyploid rice restorer lines. Both PS006 and PS012 had good outcrossing rates (Table 6). However, differences were observed between the restorer lines. Generally, PS006 had a higher outcrossing rate than PS012.

**Table 6:** Outcrossing rate of male sterile lines.

Line	Male parent	Outcrossing rate (%)	Line	Male parent	Outcrossing rate (%)
PS006	R073	71.29 ± 5.32	PS012	R057	34.53 ± 4.26
	R075	58.23 ± 3.33		R068	23.42 ± 2.57
	R076	61.45 ± 5.31		R073	30.25 ± 3.78
	A047	41.28 ± 3.69		R076	34.09 ± 4.05
	A094	57.52 ± 6.03		A077	24.03 ± 1.98
	H123	67.98 ± 4.65		H123	39.44 ± 2.79
	H156	36.59 ± 2.78		H156	37.23 ± 3.02
	ZBR018	60.36 ± 3.21		ZBR018	46.49 ± 6.05
	ZB030	49.68 ± 4.36		ZB030	40.27 ± 4.52
	ZB068	55.44 ± 2.99		ZB068	38.33 ± 3.26
	ZB176	63.57 ± 8.18		ZB176	53.56 ± 3.55

R057, R068, R073, R75, R076, A047, A077, A094, H123, H156, ZBR018, ZB030, ZB068, ZB176 are tetraploid rice restorer lines with normal fertility, which were bred by our research group.

### Analysis of the PTGMS gene background of PS006 and PS012

**Figure 4:**
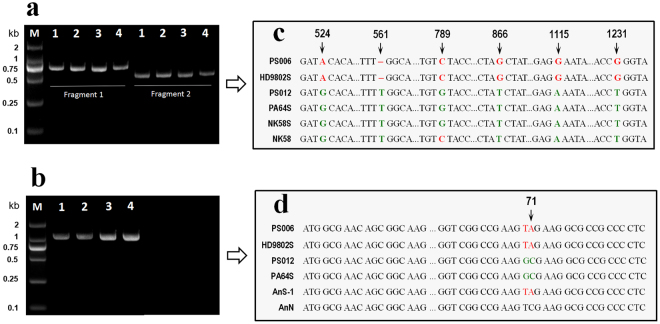
Detection of *pms3* and *tms5* genes in PS006 and PS012. (**a**) PCR product bands of *pms3* in PS006 (1), PS012 (2), HD9802S (3) and PA64S (4). M: marker 2000. Fragment 1 is the first half of the *pms3* gene (808-bp.) Fragment 2 is the second half of the *pms3* gene (638-bp). The two fragments are spliced into a full *pms3* gene (1236-bp). (**b**) PCR product bands of a 1060-bp fragment of the *tms5* gene in PS006 (1), PS012 (2), HD9802S (3) and PA64S (4). This sequence was designed to contain the target site. M: marker 2000. (**c**) Sequence comparison of the *pms3* gene in PS006, PS012, HD9802S, PA64S, NK58S and NK58. The mutation sites are marked in red. The mutated nucleotide positions (arrows) are shown at the top. (**d**) Sequence comparison of *tms5* in PS006, PS012, HD9802S, PA64S, AnS-1 and AnN. The mutated nucleotide at positions 70 and 71 (arrow) are shown in red and bold.

To test the PTGMS gene resource of PS006 and PS012, we cloned the full *pms3* gene (1236-bp) (Fig. 4a) and a 1060-bp fragment of the *tms5* gene containing the single-nucleotide polymorphism (SNP) site (Fig. 4b). A sequence comparison of the *pms3* gene in PS006, PS012, HD9802S, Pei’ai 64 S (PA64S), Nongken 58 S (NK58S) and Nongken 58 (NK58) showed that PS012 had the same sequence as PA64S and NK58S, which contained the same substitution of C–to–G (789 site) compared with NK58. The sequence of PS006 was the same as HD9802S, which contained a four-point mutation at position 524 (G-to-A), 866 (T-to-G), 1115 (A-to-G) and 1231 (T-to-G) and a single-base deletion at the 561 site (Fig. 4c). A sequence analysis of the *tms5* gene in PS006, PS012, HD9802S, PA64S, AnnongS-1 (NnS-1) and Annong (NnN) revealed that PS006 had the same sequence as HD9802S and NnS-1, which contained the mutated nucleotide (C-to-A) at position 71 compared with NnN. However, PS012 and PA64S had the same substitution of T-to-G at position 70. The results indicated that the two polyploid rice PTGMS lines had different PTGMS gene backgrounds, with PS006 carrying the TGMS gene *tms5*, which was transferred from HD9802S, and PS012 carrying the *pms3* gene, which was transferred from PA64S.

These results together suggested that PS006 and PS012 were typical two-line male sterile lines and exhibited sterility under high-temperature and long-day conditions and fertility under low-temperature and short-day conditions. These lines presented good plant types, good flowering habits, and high stigma exsertion and outcrossing rates; therefore, they appear to represent suitable materials for polyploid hybrid rice research and exploitation.

### Combining ability analysis of the main characteristics of the hybrid combinations

Eight crosses were generated according to a partial-diallel cross using the two polyploid rice PTGMS lines and four polyploid rice restorer lines. The results of a variance analysis of the eight main characteristics showed significant differences between the combinations (Table 7), which indicated genetic differences among the genotypes for these traits and genetic differences among the combinations.

**Table 7:** Variance analysis on main characters of hybrids (F-value).

Item	PP	PH	PL	FGP	TGP	SR	GW	YP
Combination	92.540**	632.023**	360.874**	141.518**	214.803**	69.956**	6.864**	58.848**
PS	7.877**	133.053**	101.896**	235.624**	228.331**	48.455**	2.243**	62.035**
PR	123.479**	720.083**	221.960**	79.216**	46.098**	106.237**	5.213**	49.867**
PS × PR	89.737**	710.286**	586.115**	172.452**	378.998**	40.841**	10.055**	66.766**

PS: PTGMS lines; PR: restorer lines; PP: Panicle number per plant; PH: Plant height; PL: Spikelet length; TGP: Total grain number per panicle; FGP: Filled grain number per panicle; SR: Seed-setting rate; GW: 1000-grain weight; YP: Grain weight per plant. **Significant at P < 0.01.

Further analysis of the general combining ability (GCA) of PS006 and PS012 was performed based on the aforementioned analysis. The GCA in the same parent differed among the eight traits (Table 8). The panicle number per plant (PP), filled grain number per panicle (FGP), total grain number per panicle (TGP), seed-setting rate (SR) and grain weight per plant (YP) of PS006 mainly showed additive effects; however, the plant height (PH), spikelet length (PL) and 1000-grain weight (GW) had negative GCA effects. The GCA values showed that PS006 was a good male parent for increasing FGP, TGP and SR and could increase the production of the F_1_ hybrids. In contrast, the GCA effects of the PH, PL and GW for PS012 were significantly positive, whereas the PP, FGP, TGP, SR and YP were negative. The hybrids of PS012 may show tall plants, long spikelets and heavy grains. Thus, high-yield characteristics of large spikelets and heavy grains could be obtained using PS012.

**Table 8:** The general combining ability values of polyploid hybrid rice combinations.

Parents	PP	PH	PL	FGP	TGP	SR	GW	YP
PS006	0.73	–2.3	–0.48	19.33	16.58	2.80	–1.13	13.74
PS012	–0.73	2.3	0.48	–19.33	–16.58	–2.80	1.13	–13.74
ZBR018	–6.64	4.50	–0.83	–0.92	–15.42	6.92	1.09	–34.97
ZB068	1.13	0.07	–0.97	9.25	18.42	–2.34	1.35	22.13
ZB167	4.31	12.55	–0.68	14.58	10.92	2.68	–3.19	20.89
ZB030	1.20	–17.12	2.47	–22.92	–13.92	–7.26	0.76	–8.05

ZBR018, ZB030, ZB068, ZB167 are tetraploid rice restorer lines with normal fertility.

To further investigate the yield level of the hybrids, we analysed the specific combining ability (SCA) of the polyploid hybrid rice combinations. The SCA values showed differences among hybrids for the same combination of different traits or the same trait with different combinations (Table 9). Additionally, the SCA effect of different combinations from the same male parent also differed, and the results indicated diverse gene interactions in polyploid hybrid rice. Thus, the SCA values can be used to guide the breeding of high-yield polyploid hybrid rice combinations.

**Table 9:** The specific combining ability values of polyploid hybrid rice combinations.

Combinations	PP	PH	PL	FGP	TGP	SR	GW	YP
PS006 × ZBR018	0.74	1.80	0.29	20.33	31.08	–2.32	–2.90	4.29
PS006 × ZB068	4.84	6.30	–0.09	14.50	22.92	–0.20	–0.41	40.14
PS006 × ZB167	–5.01	–17.12	3.36	16.50	9.42	5.58	1.08	–11.16
PS006 × ZB030	–0.56	9.02	–3.56	–51.33	–63.42	–3.06	2.23	–33.27
PS012 × ZBR018	–0.74	–1.80	–0.29	–20.33	–31.08	2.32	2.90	–4.29
PS012 × ZB068	–4.84	–6.30	0.09	–14.50	–22.92	0.20	0.41	–40.14
PS012 × ZB167	5.01	17.12	–0.36	–16.50	–9.42	–5.58	–1.08	11.16
PS012 × ZB030	0.56	–9.02	3.56	51.33	63.42	3.06	–2.23	33.27

### Heterosis and utilization potential analysis of polyploid hybrids

**Figure 5:**
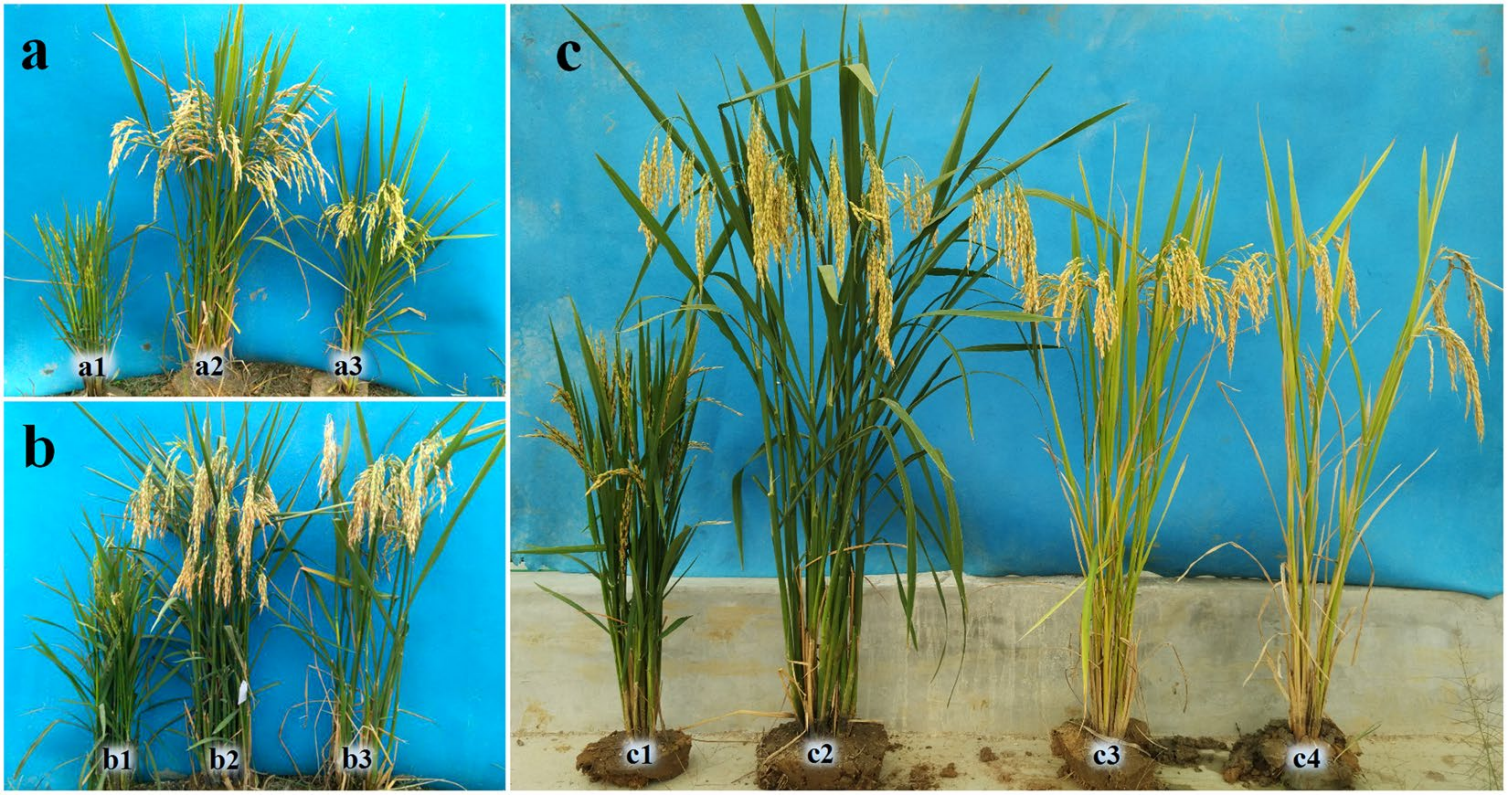
Morphological characteristics of the diploid and polyploid hybrids. (**a**) Plant appearance of PS006 (a1) and ZB030 (a3) and their F_1_ hybrid ZY008 (a2). ZB030 was a polyploid rice restorer line with normal fertility. (**b**) Plant appearance of PS012 (b1) and ZB167 (b3) and their F_1_ hybrid ZY012 (b2). ZB167 was another polyploid rice restorer line with normal fertility. (**c**) Plant appearance of PS006 (c1), XH216 (c2), Liangyou 287 (c3) and Yangdao 6 (c4). XH216 was an F_1_ hybrid of PS006. Liangyou 287 was a diploid hybrid rice variety. Yangdao 6 was a diploid cultivated rice variety.

To investigate the heterosis and potential application value of polyploid rice hybrids, we compared the main agronomic traits among polyploid hybrids, diploid hybrids and conventional rice cultivars. Compared with the parents, the polyploid hybrids had stronger growth and tillering ability (Fig. 5a and b), and they also had larger spikelets, more grains per spike and heavier grains (Tables 1 and 10). These results suggest that heterosis occurred in the F_1_ generation of the polyploid hybrid rice, which was consistent with the results of the GCA and SCA analyses. The application value of polyploid rice depends on whether it presents advantages when compared with diploid rice. Compared with the diploid hybrid rice Liangyou 287 (Early Hybrid Rice, bred by professor Zhou, Hubei University, China), the polyploid hybrid rice line XH216 had sturdier stems, which could help reduce lodging (Table 10 and Fig. 5c). In addition, studies of the yield traits showed obvious differences in the GW, with the weight of XH216 (43.69 g) nearly twice that of Liangyou 287 (24.56 g). The TGP and SR of XH216 were lower; however, the GW per plant (54.70 g) was higher than that of Liangyou 287 (37.11 g) and Yangdao 6 (33.26 g). The results indicated that, compared with their parents, the F_1_ hybrids of polyploid hybrid rice showed heterosis and that, compared with diploid rice, the F_1_ hybrids demonstrated the potential for higher rice yields.

**Table 10:** Main agronomic traits of diploid and polyploid rice.

Lines	Plant height (cm)	No. of tillers	Spikelet length (cm)	Grain length/width (cm)	Awn length (cm)	Total grain number per panicle	Seed-setting rate (%)	1000-grain weight (g)
ZB030	96.05 ± 1.25	8.10 ± 0.90	25.14 ± 1.24	0.92 ± 0.03/0.36 ± 0.02	1.36 ± 0.35	132.40 ± 8.60	79.07 ± 2.46	42.17 ± 0.13
ZB167	119.65 ± 3.47	7.91 ± 1.66	27.34 ± 2.07	0.95 ± 0.03/0.42 ± 0.01	1.93 ± 0.44	143.21 ± 9.73	81.14 ± 3.39	39.76 ± 0.57
ZY008	115.82 ± 4.69	16.33 ± 3.12	26.75 ± 1.09	0.91 ± 0.04/0.37 ± 0.02	0.96 ± 0.25	137.95 ± 4.83	83.67 ± 6.26	43.68 ± 1.05
ZY012	121.73 ± 2.35	17.56 ± 5.10	27.95 ± 2.13	0.90 ± 0.03/0.45 ± 0.02	1.53 ± 0.45	153.83 ± 5.16	85.96 ± 5.27	40.03 ± 0.42
XH216	117.79 ± 2.60	10.36 ± 4.17	28.43 ± 2.19	1.05 ± 0.03/0.40 ± 0.03	0.86 ± 0.18	142.31 ± 8.97	84.92 ± 7.04	43.69 ± 0.72
Yangdao 6	108.36 ± 4.69	7.50 ± 2.12	25.73 ± 1.09	1.03 ± 0.06/0.30 ± 0.03	0.62 ± 0.20	162.06 ± 10.33	90.78 ± 2.65	30.14 ± 0.63
Liangyou 287	102.58 ± 6.29	10.84 ± 3.86	21.77 ± 2.13	1.04 ± 0.05/0.28 ± 0.02	0.00	156.83 ± 7.92	88.89 ± 5.41	24.56 ± 0.67

ZB030 and ZB167 are tetraploid rice restorer lines with normal fertility. ZY008, ZY 012 and XH216 are F_1_ hybrids of tetraploid rice. Liangyou 287 is a diploid hybrid rice variety. Yangdao 6 is a diploid cultivated rice variety.

## Discussion

The discovery of male sterile lines plays a crucial role in the utilization of rice heterosis^1,8,28^. To explore and exploit rice heterosis at the polyploid level, we first established a breeding technology for obtaining polyploid rice PTGMS lines based on chromosome doubling, complex hybridization and self-breeding. Certain crucial parameters are required for the success of such technology. First, the parent materials of the PTGMS lines and PMeS lines are required to provide the PTGMS and PMeS genes, particularly the PMeS gene, which can promote the SRs of the polyploid rice PTGMS lines in their fertile stage. Second, the chromosome doubling frequency affects the progress of breeding. Usually, the vitality of the callus, the concentration of colchicine and the time of colchicine treatment are key factors for success. Using this technology, two polyploid rice PTGMS lines were successfully bred: PS006 and PS012.

Previous research has shown that PS006 and PS012 are tetraploid *indica* rice lines, and they present unique agronomic characteristics that are useful for rice breeding, such as strong stems, large panicles and stigmas, and long oval grains, which conform to the typical features of polyploidy. Flowering habit studies have revealed that both of these lines have good panicle uniformity, concentrated flowering periods, and good stigma exsertion rates, which would be more conducive to hybrid seed production^29^. In addition, we noticed that PS006 and PS012 had fertility alteration characteristics. Under high-temperature (above 23.6 °C for PS006 and 24.4 °C for PS012) and long-day conditions, the lines are male sterile. However, under low-temperature (below 23.6 °C for PS006, and below 24.4 °C for PS012) and short-day conditions, these lines convert to male fertile; thus, they can self-pollinate. We inferred that the fertility of these lines was mainly induced by temperature and photoperiod. In two-line hybrid breeding, the cultivation of sterile lines with low critical sterility-inducing temperature (CSIT) is a key requirement for ensuring the purity of hybrid seeds^30^, and the CSIT shows that PS006 (below 23.6 °C) would be safer than PS012 (below 24.4 °C) in two-line polyploid hybrid rice breeding. We hypothesize that the differences in CSIT are related to the different PTGMS gene backgrounds. In our study, the PTGMS genes of PS006 were transferred from HD9802S, which presents a low CSIT (<23.5 °C)^31^. HD9802S and PS006 carried the same *tms5* mutation. This mutation leads to the TGMS trait through a loss of RNase Z^S1^ function, which is responsible for processing *Ub*_L40_ mRNAs and controlling thermo-sensitive genic male sterility in rice^32^. However, the PTGMS genes of PS012 were transferred from PA64S, which is a TGMS rice line that was developed by transferring PTGMS genes from NK58S. The TGMS trait is conferred by *p/tms12-1* (*pms3*), which encodes a unique noncoding RNA that produces a 21-nucleotide small RNA^9,10^. However, the sterility gene from NK58S, in which the PGMS trait is determined by *pms1*, *pms2* and *pms3* ^10,33–35^ via a single genetic background, usually has a high CSIT^14,30^. The thermo-photoperiod sensitivity characteristics of PS012 are consistent with this conclusion. Recently, Zhou *et al*.^36^ suggested that TGMS lines with higher CSITs could be crossed with lower CSIT lines to select new TGMS lines with lower CSITs. Thus, the polyploid rice PTGMS lines with lower CSITs from PS006 and PS012 populations are selected according to this suggestion. In the present study, we also investigated the combining ability for the main agronomic traits in the hybrids generated by PS006 and PS012. We found that PS006 presented additive effects for PP, FGP, TGP, SR and total YP but had negative effects for PH, PL and GW. However, PS012 showed the opposite effects. The analysis of the combining ability indicated that high-yield polyploid hybrid rice combinations could be bred using suitable parents. Studies of the yield and yield-related traits among polyploid parents and hybrids, diploid hybrids and conventional rice have confirmed these findings. Compared with the parents, the polyploid F_1_ hybrids had high parent heterosis for the tillers per plant, TGP, SR and grain yield per plant but not for the grain length and width. These results are consistent with other studies, which also found high parent heterosis for filled grains per panicle, SR and yield but negative high parent heterosis for grain length and width in polyploid rice^14–16,21^. Researchers have inferred that a complex genetic mechanism controls the heterosis in polyploid rice, and many genes related to fertility and heterosis in autotetraploid rice have been found^37–39^. The complex regulatory mechanisms might soon be revealed^14,40^. Our work suggests that PS006 and PS012 represent suitable material for further studies of polyploidy and hybrid vigour in rice.

In summary, the findings reported in this study provide new germplasm data for rice research and insights for studies of the two-line hybrid system of polyploid rice.

## Methods

### Plant materials

The PS006 line was selected from four rice lines: HD9802S-2*x* (*O. sativa* ssp*. indica*, 2n = 2*x* = 24), which is a PTGMS line presented by Professor Yong Zhou (Hubei University, China); HD9802S-4*x* (*O. sativa* ssp*. indica*, 2n = 4*x* = 48), which was bred by our research group; and HN2026-2*x* (*O. sativa* ssp*. japonica*, 2n = 2*x* = 24) and HN164-4*x* (*O. sativa* ssp*. japonica*, 2n = 4*x* = 48), which were bred by our research group and with the *PMeS* gene. The line PS012 was also selected from four rice lines: PA64S-2*x* (*O. sativa* ssp*. indica*, 2n = 2*x* = 24), which is a PTGMS line presented by Academician Longping Yuan (Hunan Hybrid Rice Research Centre, China); PA64S-4*x* (*O. sativa* ssp*. indica*, 2n = 4*x* = 48) and A175-4*x* (*O. sativa* ssp*. japonica*, 2n = 4*x* = 48), which were bred by our research group using the *PMeS* gene; and HN2026-2*x*.

### Breeding procedures

**Figure 6:**
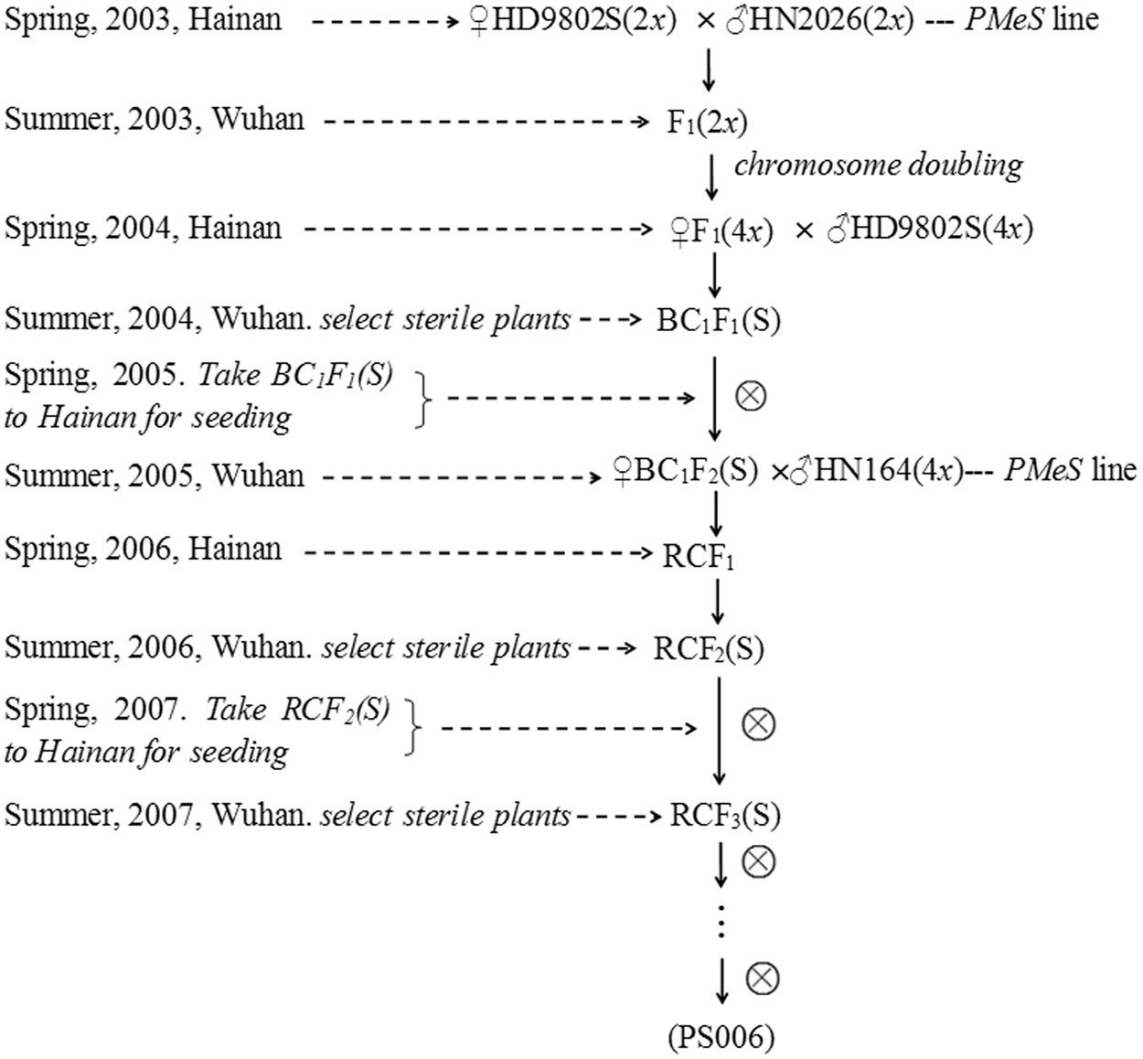
Breeding process of PS006.

The breeding process of PS006 and PS012 included parental selection, cross and composite cross, chromosome doubling, polyploid identification, fecundity identification and self-crossing to produce stable lines. PS006 is used as an example (Fig. 6).

(1) Parental selection. In the breeding of PS006, the parent materials were HD9802S-2*x*, HN2026-2*x*, HD9802S-4*x* and HN164-4*x*. (2) Cross. During flowering, HD9802S-2*x* was used as the female parent in crosses with HN2026-2*x*. (3) Chromosome doubling. The method used was modified according to the reports of Liu^41^ and Li^42^. The F_1_ (2*x*) of HD9802S and HN2026 was planted in the field. In the stage of panicle differentiation, young panicles of F_1_ were cultured on an N6 solid medium to induce callus. Approximately 3 weeks later, the vigorous calli were transferred to the liquid medium with 500–750 mg l^−1^ colchicine for chromosome doubling. After a recovery culture process, the calli treated with colchicine were placed in a differentiation medium to form buds. The shoots were induced to produce roots on a 1/2 MS medium. Plantlets were transferred to the field and allowed to grow into whole rice plants. (4) Polyploid identification. In the breeding process, the synthetic autotetraploid rice HD9802S-4*x*, PA64S-4*x* and F_1_-4*x* and the polyploid rice PTGMS line PS006 were identified via morphological observations and the root tip chromosome number. (5) Backcross and sterile plant selection. The identified tetraploid F_1_ (♀) was hybridized with HD9802S-4*x* (♂). Then, sterile plants were selected from the BC_1_F_1_ in Wuhan (under high-temperature and long-day conditions) and then transported to Hainan to identify their fertility under low-temperature and short-day conditions and allowed to produce seeds (BC_1_F_2_). (6) Composite cross and sterile plant selection. BC_1_F_2_ (♀) was crossed with HN164-4*x* (♂) to obtain RC_1_F_1_. These RC_1_F_2_ plants were planted in Wuhan. Then, sterile plants were selected from the RC_1_F_2_ plants in Wuhan and transported to Hainan to identify their fertility. (7) Self-crossing for line stability. The plants selected from the PS006 lines were self-crossed and formed stable lines, and their final line numbers matched the original numbers at initial selection (BCF_2_), i.e., PS006.

The breeding procedure for PS012 was similar to that for PS006; however, the parent materials were PA64S-2*x*, HN2026-2*x*, PA64S-4*x* and A175-4*x*.

### Chromosome identification and morphological observations

Plants from the synthetic autotetraploid rice HD9802S-4*x*, PA64S-4*x* and F_1-_4*x* and the polyploid rice PTGMS lines PS006 and PS012 were examined by counting the chromosome numbers in their root tips according to the methods of Li^42^. The observations and photographic recordings were performed using an Olympus BX51 microscope (made in Japan). The key morphological traits PH, spikelet number, PL, grain length and width, awn length, shattering trait, seed colour and seed set were investigated. The recording methods and standards were set according to the protocols of Gai^43^.

### Identification of *indica-japonica* attributes

The *indica-japonica* attributes of the PS006 and PS012 lines were identified by 19 pairs of insertion/deletion (InDel) molecular markers designed based on the comparative genomic DNA sequences between *indica* variety 9311 and *japonica* variety Nipponbare. The method of Lu^44^ was modified to detect the *indica/japonica* gene frequency using InDel molecular markers, and the tested materials were evaluated to determine differences in their attributes, e.g., *indica*, *indica*-cline, intermediate *indica*-cline, intermediate *japonica*-cline, *japonica*-cline and *japonica* types. The formulae for the calculations are as follows: gene frequencies of *indica*, $Fi=\frac{2{\sum }_{1}^{N}Xii+{\sum }_{1}^{N}Xij}{2N}$; and gene frequencies of *japonica*, $Fj=\frac{2{\sum }_{1}^{N}Xjj+{\sum }_{1}^{N}Xij}{2N}$, where X*ii* is the *indica* homozygous genotype II, X*jj* is the *japonica* homozygous genotype JJ, X*ij* is the *indica-japonica* heterozygous genotype IJ, and N is the number of InDel molecular markers.

### PTGMS gene background investigation

To investigate the PTGMS genes of PS006 and PS012, the sequences of *pms3* (1236-bp) and *tms5* (a 1060-bp fragment) were amplified via PCR. Genomic DNA from the leaves of plants was extracted using the sodium dodecyl sulphate method^45^. PCR amplifications were performed using two specific primers for the *pms3* gene (primer 1, F: 5′-ggcatgtgtcttagggttttta-3′, R: 5′-accatgcctcccactcctatat-3′; and primer 2, F: 5′-aagcagagacatagatgagcaaca-3′, R: 5′-agcctatgtttcttctgccttg-3′), and one specific primer for the *tms5* gene (F: 5′-tggccaaacagctgctacttca-3′, R: 5′-atggcgtggtaggtcttgaagg-3′) surrounding the designed target sites. The PCR products were purified and then directly sequenced (by the Tsingke biological technology company, Beijing, China). The sequences were analysed using BLAST (http://www.ncbi.nlm.nih.gov/BLAST/).

### Flowering habit studies

The stigma exsertion characteristics, flowering duration of a single spikelet and flowering distribution of spikelets on a single panicle were investigated.

### Fertility alteration research

The characteristics of fertility alteration of the PS006 and PS012 lines were experimentally studied by sowing rice by stage, breeding in a phytotron and observing the pollen fertility under natural conditions in both Wuhan and Hainan. Pollen fertility was determined as the percentage of pollen grains stained with 1% I_2_-KI and observed under an optical microscope.

### Combining ability analysis

The polyploid rice PTGMS lines PS006 and PS012 were crossed with four high seed-setting polyploid rice restorer lines in Wuhan. Then, all parent lines and their F_1_ progeny were planted in Hainan. When the materials were mature, five representative plants of each type were randomly selected, and their main agronomic characteristics were investigated as follows: tiller number, PH, PL, grain number and GW. Then, the GCA and SCA values of the polyploid hybrid rice combinations were analysed using SPSS and Microsoft Excel.

### Polyploid hybrids and diploid rice comparison

The main morphological traits of the polyploid hybrids and diploid hybrid and conventional rice cultivars were compared.
